# Hepatic Deletion of Smad7 in Mouse Leads to Spontaneous Liver Dysfunction and Aggravates Alcoholic Liver Injury

**DOI:** 10.1371/journal.pone.0017415

**Published:** 2011-02-28

**Authors:** Lu Zhu, Lingdi Wang, Xiao Wang, Xiaolin Luo, Ling Yang, Rui Zhang, Hongkun Yin, Dong Xie, Yi Pan, Yan Chen

**Affiliations:** Key Laboratory of Nutrition and Metabolism, Institute for Nutritional Sciences, Shanghai Institutes for Biological Sciences, Graduate School of the Chinese Academy of Sciences, Chinese Academy of Sciences, Shanghai, China; Institut Pasteur of Shanghai, China

## Abstract

**Background:**

TGF-β has been known to play an important role in various liver diseases including fibrosis and alcohol-induced fatty liver. Smad7 is an intracellular negative regulator of TGF-β signaling. It is currently unclear whether endogenous Smad7 has an effect on liver function and alcoholic liver damage.

**Methodology/Principal Findings:**

We used Cre/loxP system by crossing Alb-Cre mice with Smad7^loxP/loxP^ mice to generate liver-specific deletion of Smad7 with loss of the indispensable MH2 domain. Alcoholic liver injury was achieved by feeding mice with a liquid diet containing 5% ethanol for 6 weeks, followed by a single dose of ethanol gavage. Deletion of Smad7 in the liver was associated with increased Smad2/3 phosphorylation in the liver or upon TGF-β treatment in primary hepatocytes. The majority of mice with liver specific deletion of Smad7 (Smad7^liver-KO^) were viable and phenotypically normal, accompanied by only slight or no reduction of Smad7 expression in the liver. However, about 30% of Smad7^liver-KO^ mice with high efficiency of Smad7 deletion had spontaneous liver dysfunction, demonstrated as low body weight, overall deterioration, and increased serum levels of AST and ALT. Degeneration and elevated apoptosis of liver cells were observed with these mice. TGF-β-induced epithelial to mesenchymal transition (EMT) was accelerated in Smad7-deleted primary hepatocytes. In addition, alcohol-induced liver injury and steatosis were profoundly aggravated in Smad7 deficient mice, associated with upregulation of critical genes involved in lipogenesis and inflammation. Furthermore, alcohol-induced ADH1 expression was significantly abrogated by Smad7 deletion in hepatocytes.

**Conclusion/Significance:**

In this study, we provided *in vivo* evidence revealing that endogenous Smad7 plays an important role in liver function and alcohol-induced liver injury.

## Introduction

Liver dysfunction is a life-threatening medical scenario that demands clinical care. Severe liver dysfunction leads to liver failure that occurs when the majority of liver tissue is damaged beyond repair and the liver is no longer able to perform normal functions [1]. In most cases, liver dysfunction occurs gradually over many years. However, a rare condition known as acute liver failure such as fulminant hepatitis can occur rapidly. Transforming growth factor-β ( (TGF-β) plays an important role in liver diseases [2]. TGF-βs belong to a large family of growth and differentiation factors that utilize complex signaling networks to regulate numerous cellular activities including differentiation, proliferation, motility, adhesion, and apoptosis [3]. The TGF-β family members regulate gene expression via serine/threonine kinase receptors at the cell surface and a group of intracellular transducers called Smad proteins including R-Smads (receptor-specific Smad, including Smad1, 2, 3, 5 and 8), Co-Smad or Smad4 (a common-Smad), and I-Smads (inhibitory Smads, including Smad6 and Smad7) [3], [4], [5], [6]. The signaling starts by binding of the ligand to the cognate transmembrane receptor kinase, followed by phosphorylation of R-Smad and complex formation between R-Smad with Co-Smad. The Smad complex transduces the signal from the plasma membrane into the nucleus in which Smad proteins and their transcriptional partners directly regulate gene expression [3], [6]. Smad7 is a member of the I-Smad subfamily that is able to directly interact with the TGF-β type I receptor [7], whereas blocking the phosphorylation of R-Smads Smad2 and Smad3 and inhibiting TGF-β signaling.

Alterations in the production of TGF-β or mutations within the genes involved in TGF-β signaling pathway are associated with the pathogenesis of many diseases including fibrotic disease of the kidney, liver and lung. The *in vivo* functions of the Smad proteins as well as their association with diseases are revealed by targeted deletion of the corresponding genes in mice [8]. Deletions of Smad1, Smad2 and Smad4 lead to embryonic lethality of the mouse, indicating the importance of these genes in early development [9], [10], [11]. Deletion of Smad3 gives rise to abnormalities in mucosal immune system, related to development of colorectal cancers [12], [13]. Mouse deletion studies also indicate that Smad5 is involved in angiogenesis during embryogenesis [14]. A recent *in vivo* study indicates that Smad8 is involved in pulmonary vascular remodeling [15]. Interestingly, deletion studies of inhibitory Smads suggest that both Smad6 and Smad7 are involved in cardiovascular development in the mouse. Deletion of the indispensable MH2 domain of Smad6 results in multiple cardiovascular defects during early development [16]. On the other hand, deletion of the MH2 domain of Smad7 leads to defects in the development of atrioventricular cushion [17], while hypomorphic Smad7 deficiency with deletion of the MH1 domain of Smad7 is associated with altered B-cell response [18].

A few studies have emerged to reveal the role of Smad7 in liver diseases. Overexpression of Smad7 in mouse liver could attenuate TGF-β signaling and improve carbon tetrachloride (CCl_4_)-provoked liver fibrosis [19]. On the other hand, hypomorphic Smad7 deficiency enhances CCl_4_-induced liver damage and fibrosis [20]. In this study, we established a mouse model with liver-specific deletion of the MH2 domain of Smad7. Interestingly, we found that deletion of Smad7 is associated with development of spontaneous liver dysfunction in the mouse.

The most common causes of chronic liver injury include virus infection, long term alcohol consumption, cirrhosis, inherited disorders, and malnutrition. Among these major factors that cause chronic liver injury, alcohol drinking is a major etiologic one in chronic liver disease worldwide, causing fatty liver, alcoholic hepatitis, cirrhosis, and eventually hepatocellular carcinoma. In the past few decades, major progress has been made in our understanding about the molecular mechanisms underlying alcoholic liver injury, such as the functional roles of STAT3 [21]. We also found that Smad7 deficiency is able to enhance formation of alcohol-induced fatty liver. These results, combining with the studies from other laboratories, pinpoint an important role of Smad7 in liver functionalities and liver diseases.

## Results

### Generation and characterization of liver-specific Smad7 deletion mouse

**Figure 1:**
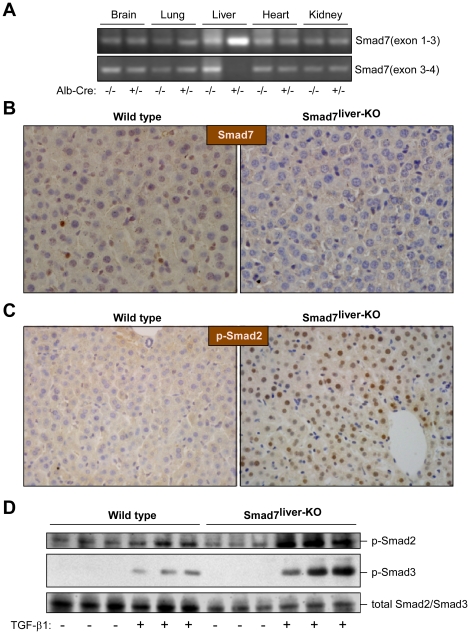
Characterization of Smad7^liver-KO^ mice. (A) Liver-specific deletion of Smad7. RT-PCR analysis was performed with total RNA isolated from multiple tissues in Alb-Cre heterozygous or wild type mice (all mice having Smad7^loxP/loxP^) with specific primers that amplify mRNA regions corresponding to exons 1–3 or exons 3–4 of Smad7 respectively. (B) Analysis of the Smad7 by immunohistochemistry staining. Representative liver sections (400 X) from wild type and Smad7^liver-KO^ mice were used in immunohistochemistry staining with an anti-Smad7 antibody. The nuclei were stained with haematoxylin. (C) Smad2 phosphorylation is elevated in the liver of Smad7^liver-KO^ mice. Smad2 phosphorylation was analyzed by immunohistochemistry using liver sections from either wild type or Smad7^liver-KO^ mice. The nuclei were stained with hematoxylin. Note that nuclear phospho-Smad2 staining is increased in the liver of Smad7^liver-KO^ mice. (D) TGF-β-induced Smad2 and Smad3 phosphorylation is enhanced by Smad7 deletion in primary hepatocytes. Immunoblotting was performed using total protein lysate extracted from primary hepatocytes using antibodies as indicated. The cells were treated with or without 5 ng/ml of TGF-β1 for 24 hours as indicated after overnight serum starvation.

To investigate the potential function of Smad7 in the liver, we crossed Albumin-Cre transgenic mice with Smad7^loxP/loxP^ mice that contain two loxP fragments flanking the 5′ half of exon 4 of Smad7 gene [17]. The Albumin-Cre transgenic mice specifically express *Cre* recombinase in hepatocytes under control of a rat albumin promoter/enhancer. Specific deletion of the MH2 domain (encoded by the 5′ half of exon 4) of Smad7 in the mouse liver was confirmed by RT-PCR (Figure 1A). As expected, we found that the mRNA region corresponding to exon 1–3 was not deleted in the liver-specific Smad7-deleted mouse (Smad7^liver-KO^ mouse). However, only the mRNA region corresponding to exon 3–4 was lost in the liver of Smad7^liver-KO^ mouse, but not changed in the other tissues such as brain, lung, heart, and kidney, indicating liver-specific deletion of Smad7 MH2 domain. To verify Smad7 deletion, the protein level of Smad7 was also examined by immunohistochemistry staining in the liver sections. The result revealed that the protein level of Smad7 was markedly decreased in the liver of Smad7^liver-KO^ mouse in comparison with wide type animals (Figure 1B).

As the major cellular function of Smad7 is to inhibit TGF-β signaling, we analyzed whether deletion of Smad7 is associated with an enhancement of Smad2/3 phosphorylation in the mouse liver. By immunohistochemistry using liver sections from either wild type or Smad7^liver-KO^ mice, we found that Smad2 phosphorylation was significantly increased in the liver from the Smad7^liver-KO^ mice in comparison with the wild type animals (Figure 1C). Furthermore, we analyzed the phosphorylation levels of Smad2 and Smad3 using cultured primary hepatocytes isolated from the mice. TGF-β1-induced Smad2 and Smad3 phosphorylation appeared to be enhanced by Smad7 deletion by an immunoblotting assay (Figure 1D). Collectively, these results indicate that Smad7 deficiency is associated with enhancement of TGF-β signaling in the liver, consistent with the notion that Smad7 is an intracellular inhibitory protein to negatively modulate TGF-β signaling pathway [7].

### Deletion of Smad7 causes spontaneous liver dysfunction in the mouse

**Figure 2:**
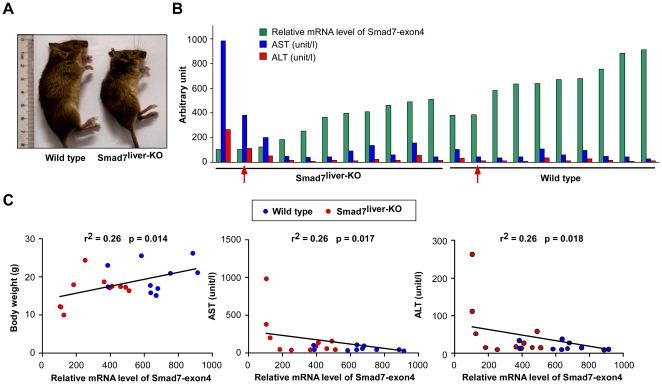
Deletion of Smad7 causes spontaneous liver failure. (A) Representative images of 12-week-old wild type and Smad7^liver-KO^ mice. (B) Relative mRNA level of Smad7 exon4 (green) as comparison with the serum AST (blue) and ALT (red) values. The arrows indicate the mice that were used in histological and immunohistochemical analyses performed in Figure 3. (C) Correlation analyses of body weight, AST and ALT levels with the mRNA level of Smad7 exon4. The values of body weight, AST and ALT values were plotted against the relative mRNA level of Smad7 exon4 for each mouse (red for Smad7^liver-KO^ mice and blue for wild type mice) and subjected to linear regression analysis. n = 11 for Smad7^liver-KO^ and n = 10 for wild type mice.

Interestingly, the phenotype of Smad7^liver-KO^ mice varied from mouse to mouse. While the majority of Smad7^liver-KO^ mice had no apparent phenotypical change, a small portion of the mice had obvious growth retardation and the overall condition started to deteriorate at 2–3 months of age (Figure 2A). We monitored the changes of liver enzymes together with the mRNA level of Smad7 corresponding to the region encoded by exon 4 in the liver (Figure 2B). In comparison with the wild type controls, ∼30% of Smad7^liver-KO^ mice had over 50% of reduction of the mRNA level of Smad7 exon 4, while ∼70% Smad7^liver-KO^ mice had only slight or no reduction of Smad7 exon 4 mRNA. The variation of Smad7 deficiency observed in the study is likely caused by incomplete expression of Cre recombinase driven by the albumin promoter (Figure S1). Interestingly, only those Smad7^liver-KO^ mice with significant deletion of Smad7 had severely decreased body weight (Figure 2C, left panel), accompanied by robustly elevated blood levels of aspartate aminotransferase (AST) and alanine aminotransferase (ALT) (Figure 2B and 2C). The blood levels of both AST and ALT were inversely correlated with the mRNA level of Smad7 exon 4 (Figure 2C, middle and right panels). Together, these data indicate that marked deletion of Smad7 expression is associated with spontaneous liver dysfunction in the mouse.

### Deletion of Smad7 increases apoptosis in hepatocytes

**Figure 3:**
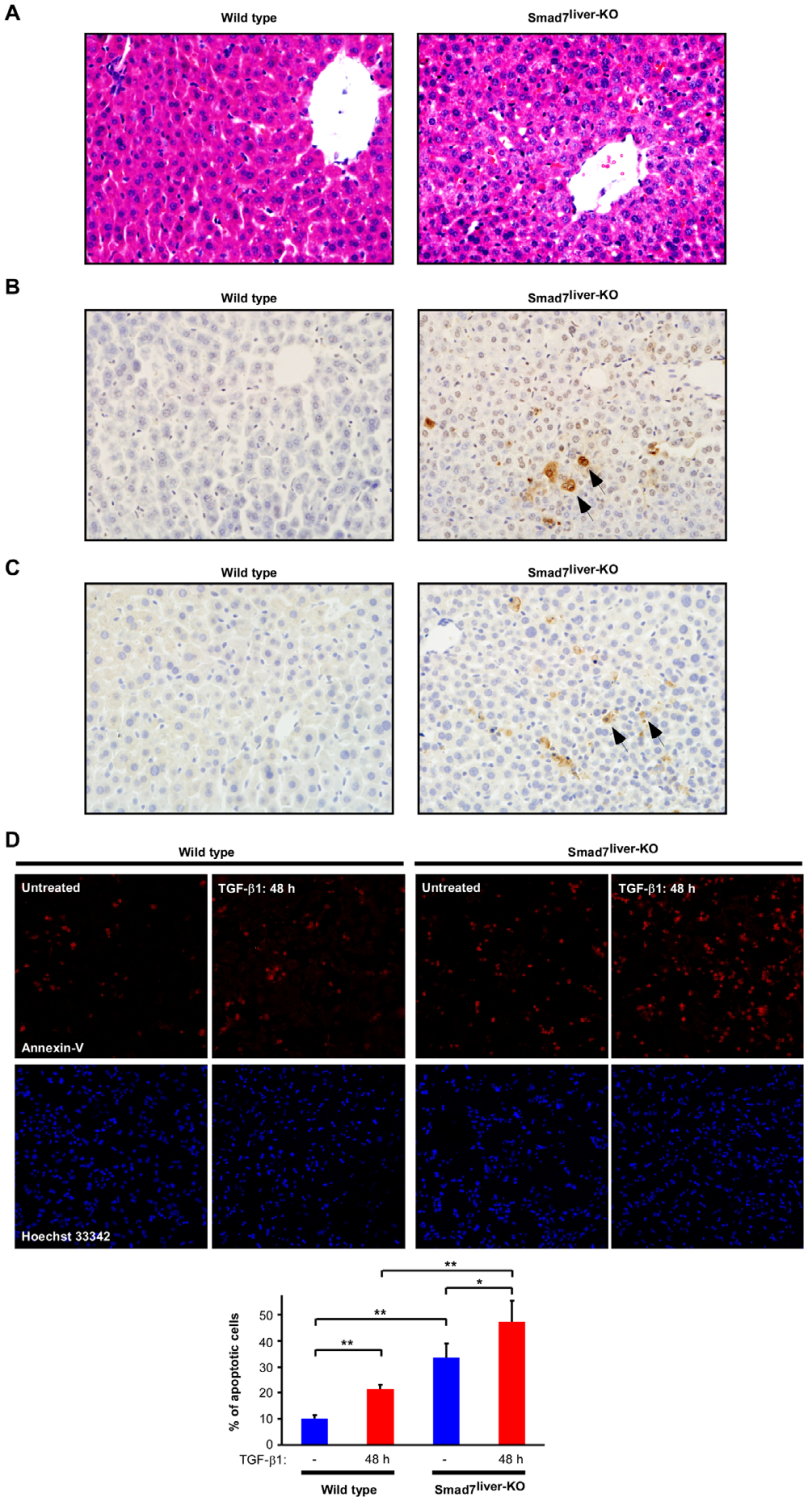
Deletion of Smad7 leads to liver degeneration and apoptosis. (A) Representative images of H&E staining of liver sections are shown with the magnification at X 400. (B) TUNEL assay of liver sections with methyl green as a counter-stain. Representative apoptotic cells are marked by arrows. Note that the Smad7^liver-KO^ mouse (with liver failure) displayed an increase of apoptosis-positive cells. (C) Immunohistochemistry with an antibody against cleaved caspase-3 to identify apoptotic cells in the liver sections. The arrows indicate apoptotic cells in the liver of Smad7^liver-KO^ mouse. (D) TGF-β-induced apoptosis is enhanced by Smad7 deletion in hepatocytes. Primary hepatocytes were treated without or with TGF-β1 (5 ng/ml) for 48 h as indicated, followed by immunofluorescence staining with Annexin V for apoptosis (Red) and Hoechst 33342 for nuclei (blue). Quantitation of the percentage of apoptotic cells in each group is shown in the bottom panel as mean ± SD. * indicate p<0.05 and ** for p<0.01 as comparison between the groups as indicated by Student's t-test.

The observed spontaneous liver damage in Smad7^liver-KO^ mice was further investigated by histological and immunohistochemical analyses. In comparison with the wild type littermate, the liver sections from the Smad7^liver-KO^ mouse with significant reduction of Smad7 expression had features of cell degeneration (Figure 3A), accompanied by elevation of apoptosis (Figure 3B and 3C, marked by arrows). Furthermore, we analyzed TGF-β-induced apoptosis in primary hepatocytes isolated from the wild type and Smad7^liver-KO^ mice. TGF-β1 treatment itself could increase the number of apoptotic hepatocytes (Figure 3D, left panel). However, the TGF-β1-induced hepatocyte apoptosis was significantly enhanced by Smad7 deletion (Figure 3D, right panel). Collectively, these results indicate that deletion of Smad7 in the liver is able to induce apoptosis of hepatocytes, leading to spontaneous liver failure in the mouse.

### Smad7 deficiency enhances TGF-β-induced EMT in hepatocytes

**Figure 4:**
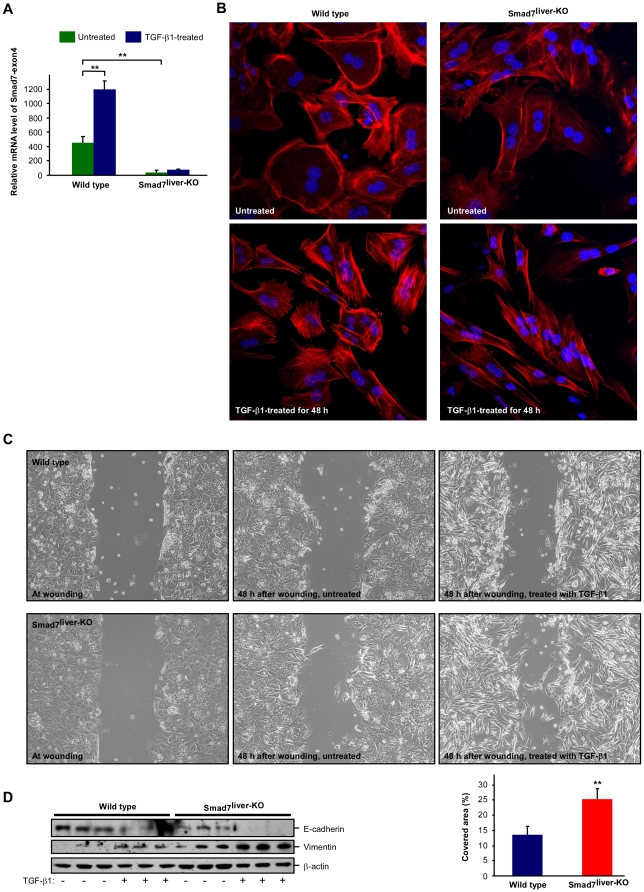
Deletion of Smad7 enhances TGF-β-induced EMT. (A) Confirmation of Smad7 deletion in primary hepatocytes isolated from Smad7^liver-KO^ mouse. Real time RT-PCR was performed with total RNA isolated from wild type or Smad7^liver-KO^ mice with primers to detect the mRNA region corresponding to exon4 of Smad7 gene. The data are shown as mean ± SD and ** indicates p<0.01 as comparison between the groups as indicated by Student's t-test. (B) TGF-β-induced EMT-like morphology changes. Immunofluorescence labeling were performed with wild type or Smad7^liver-KO^ hepatocytes treated with or without TGF-β1 (5 ng/ml) for 48 h. F-actin was stained with fluorescein isothiocyanate-labeled phalloidin (Red) and the nuclei were labeled by Hoechst 33342 (Blue). (C) Analysis of cell motility by a wound-healing assay. Cultured primary hepatocytes were analyzed by phase contrast microscopy. The cells were treated with or without TGF-β1 (5 ng/ml) for 48 h. Quantitation of the cell motility is shown in lower right panel as mean ± SD and ** indicates p<0.01 by Student's t-test. (D) Analysis of EMT markers E-cadherin and vimentin. Primary hepatocytes were treated with or without TGF-β1 (5 ng/ml) for 48 h and the cell lysate was used in immunoblotting with the antibodies as indicated.

We next analyzed the cellular function of Smad7 on epithelial to mesenchymal transition (EMT) in hepatocytes as TGF-β plays a pivotal role in EMT [22]. We isolated primary hepatocytes from wild type and Smad7^liver-KO^ mice. The cultured hepatocytes isolated from Smad7^liver-KO^ mice did have significant reduction of the mRNA encoded by Smad7 exon 4 (Figure 4A), confirming that Smad7 was successfully deleted in these cells. When the cultured primary hepatocytes were treated with TGF-β1, the cells underwent morphological changes characteristic of EMT (Figure 4B). Untreated hepatocytes exhibited a cuboidal phenotype, while TGF-β1 treatment induced a fibroblastic transition resulting in elongated and spindle-like cell morphology. Interestingly, the TGF-β1-induced EMT morphology was robustly enhanced by Smad7 deletion (Figure 4B, right panel). We also analyzed the cell motility using standard scratch-wound assays as previously described [23]. At 48 h after wounding, the untreated cells from both wild type and Smad7-deleted mice were unable to migrate into the wound area (Figure 4C). TGF-β1 treatment was able to induce migration of the cells and such effect was significantly accelerated when Smad7 was deleted (Figure 4C). TGF-β-induced EMT was further analyzed by immunoblotting to detect expression of E-cadherin and vimentin, two well-recognized markers for EMT [22]. We found that TGF-β1-induced reduction of E-cadherin and increase of vimentin was profoundly enhanced by Smad7 deletion (Figure 4D). These data, therefore, reveal that Smad7 deficiency is able to enhance TGF-β-induced EMT in hepatocytes.

### Smad7^liver-KO^ mice are more susceptible to alcohol-induced liver injury and steatosis than wild type mice

**Figure 5:**
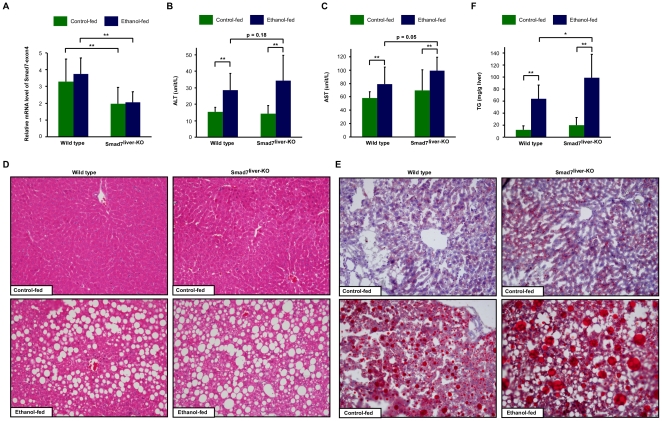
Alcohol-induced liver injury and steatosis were aggravated in Smad7^liver-KO^ mice. (A) Confirmation of Smad7 deletion. Wild type and Smad7^liver-KO^ mice were fed with control or ethanol-containing diet for 6 weeks, followed by a single gavage of 10% ethanol or maltose respectively (8 mice per group). Real time RT-PCR was performed with total RNA isolated from the mice with primers to detect the mRNA region corresponding to exon4 of Smad7 gene. (B) and (C) Measurement of serum ALT and AST. The data are shown as mean ± SD and ** indicates p<0.01 by Student's t-test. (D) Histological analysis of the liver. Representative images of H&E staining are shown for each group of mice. Please note that fatty liver degeneration induced by alcohol administration is enhanced in Smad7^liver-KO^ mice. (E) Oil-Red-O staining of the liver. (F) Triglyceride level of the liver. The data are shown as mean ± SD. * and ** indicates p<0.05 and p<0.01 respectively.

We further investigated the potential function of Smad7 deletion on alcohol-induced liver damage. Both the wild type and Smad7^liver-KO^ mice at 10 to 12 weeks old were fed with a liquid diet containing 5% ethanol or a control diet for up to 6 weeks. On the day of animal sacrifice, a single dose of gavage with 10% ethanol or isocaloric maltose dextrin was administered. We first analyzed the expression level of mRNA encoded by exon 4 of Smad7 gene to confirm that Smad7 expression was indeed significantly reduced in the Smad7^liver-KO^ mice (Figure 5A). We tracked the alteration of food intake and body weight for the entire 6-week period and found that there were no significant differences among the four groups in food intake (data not shown), except for a slightly reduced body weight gain in Smad7^liver-KO^ mice (Table 1). Alcohol exposure significantly increased the liver/body weight ratio in both wild type and Smad7^liver-KO^ mice (Table 1). Alcohol exposure decreased the levels of serum triglyceride and cholesterol in wild type mice, while Smad7-deleted mice only had a significant reduction of serum triglyceride but not cholesterol after alcohol administration (Table 1). As expected, alcohol exposure could increase serum ALT and AST activities in the mice (Figure 5B and 5C). However, the alcohol-induced raise of these enzymes was more significant in Smad7^liver-KO^ mice than the wild type animals (Figure 5B and 5C).

**Table 1:** Physiological and serum parameters of mice upon chronic-binge alcohol exposure.

Parameters	Wild type		Smad7^liver-KO^	
	Control-fed	Ethanol-fed	Control-fed	Ethanol-fed
**Body weight-initial (g)**	23.68±2.65	23.74±3.04	23.46±2.35	22.58±2.03
**Body weight-end (g)**	24.85±2.78	24.28±4.20	26.61±4.24	23.13±2.65
**Body weight-gain (g)**	1.18±2.59	0.54±2.24	3.15±2.21	0.55±1.71$$
**Liver weight (g)**	0.93±0.10	1.11±0.24	0.98±0.18	1.09±0.11
**Liver weight/body weight (%)**	3.70±0.34	4.64±0.63##	3.61±0.54	4.80±0.56$$
**EWAT (g)**	0.65±0.28	0.49±0.24	0.71±0.32	0.56±0.20
**EWAT/body weight (%)**	2.52±0.88	1.95±0.72	2.52±0.77	2.40±0.67
**Blood triglyceride (mg/ml)**	0.73±0.27	0.45±0.22#	0.80±0.35	0.47±0.10$
**Blood cholesterol (mmol/L)**	2.41±0.50	1.72±0.22##	2.17±0.52	1.89±0.47

EWAT: Epididymal white adipose tissue.

The data are shown as means ± SD (n = 8 for each group).

# and ^##^: comparison between control-fed and ethanol-fed wild type mice. ^#^ for p<0.05 and ^##^ for p<0.01.

$ and ^$$^: comparison between control-fed and ethanol-fed Smad7^Liver-KO^ mice. ^$^ for p<0.05 and ^$$^ for p<0.01.

We also analyzed the histological changes of the liver. As shown in Figure 5D and 5E, H&E staining and Oil-Red-O staining revealed that hepatic steatosis was induced by chronic alcohol exposure. Furthermore, the alcohol-induced liver steatosis was profoundly enhanced by Smad7 deletion. Consistently, Smad7 deletion led to a significant increase in the content of triglyceride level in the liver upon alcohol exposure (Figure 5F). Together, these data suggest that the liver injury and steatosis induced by chronic alcohol administration were enhanced by Smad7 deletion, further indicating that Smad7 deletion has a deteriorating effect on liver functions.

### Smad7 deficiency reduces alcohol-induced ADH1 expression in hepatocytes

**Figure 6:**
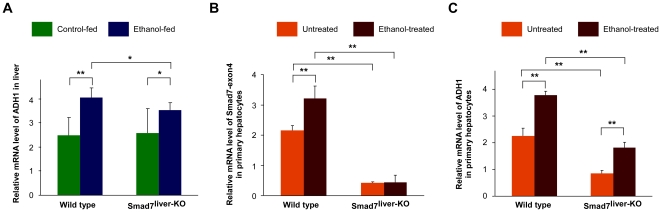
Alcohol-induced ADH1 expression is reduced by Smad7 deletion. (A) Analysis of ADH1 mRNA level of the mouse liver as of Figure 5A by real-time PCR. The data are shown as mean ± SD. * and ** indicates p<0.05 and p<0.01 respectively. (B) and (C) Analyses of the mRNA levels of Smad7 and ADH1 in primary hepatocytes. Primary hepatocytes isolated from wild type and Smad7^liver-KO^ mice were incubated with or without ethanol (100 mmol/L) for 24 h. The mRNA were isolated and used in real-time PCR. The data are shown as mean ± SD with * for p<0.05 and ** for p<0.01 between the groups as indicated.

Recently, it was reported that over-activation of TGF-β signaling may enhance alcohol-mediated liver damage by reducing expression of alcohol dehydrogenase 1 (ADH1) [24]. In wild type mice, alcohol administration significantly increased the mRNA level of ADH1 in the liver (Figure 6A). Interestingly, alcohol-induced ADH1 upregulation in the liver was slightly reduced in Smad7-deleted mice (Figure 6A). To further confirm the effect of Smad7 deletion on ADH1 expression, we isolated primary hepatocytes from the wild type and Smad7^liver-KO^ mice. The level of mRNA region corresponding to exon 4 of Smad7 gene was significantly reduced in Smad7-deleted hepatocytes, confirming that Smad7 was deleted in these cells (Figure 6C). Alcohol treatment could significantly elevate Smad7 expression (Figure 6B). Furthermore, alcohol administration could stimulate the expression of ADH1 in hepatocytes (Figure 6C). However, the expression level of ADH1 was significantly reduced in Smad7-deleted hepatocytes under both basal and alcohol-treated conditions (Figure 6C), further indicating that Smad7 deletion can reduce AHD1 expression in the liver. As Smad7 deletion is associated with activation of TGF-β signaling (Figure 1), our observation is also constant with the hypothesis that hyperactivity of TGF-β signaling aggravates alcohol-mediated liver injury through downregulation of ADH1 [24].

### Upregulation of lipogenesis- and inflammation-related genes in Smad7^liver-KO^ mice

**Figure 7:**
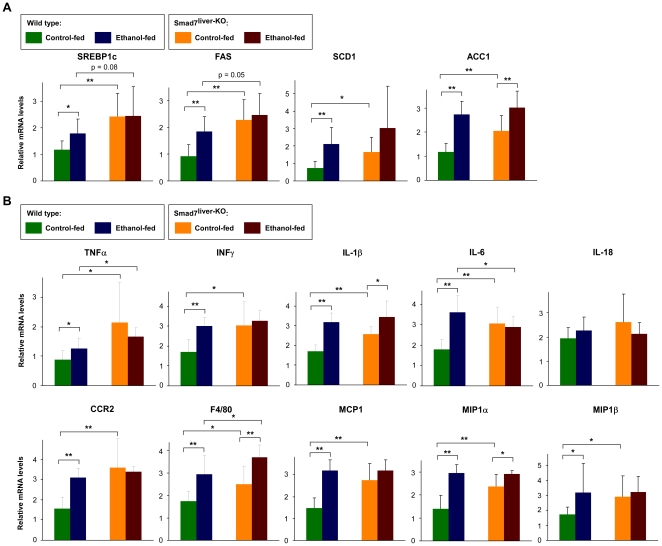
Upregulation of lipogenesis- and inflammation-related genes by ethanol treatment and Smad7 deletion. (A) Analysis of the mRNA levels of critical lipogenic genes of the mouse liver by real-time RT-PCR. Alcohol-feeding of the mice was as described in Figure 5A. The data are shown as mean ± SD. * and ** indicates p<0.05 and p<0.01 respectively. (B) Analysis of the mRNA levels of a series of inflammation-related factors by real-time RT-PCR with the samples as in Figure 5A. The data are shown as mean ± SD with * for p<0.05 and ** for p<0.01 between the groups as indicated.

It was previously reported that SREBP1c, a key regulator of fatty acid synthesis, is implicated in the development of fatty liver [25], [26]. Intriguingly, we found that the expression levels of SREBP1c as well as the critical lipogenic genes controlled by SREBP1c (including fatty acid synthase, stearoyl-CoA desaturase 1, and acetyl-CoA carboxylase-1) were all upregulated by ethanol treatment and by Smad7 deletion (Figure 7A). These results not only indicate that SREBP1c pathway is involved in ethanol-induced hepatic steatosis, but also suggest that Smad7 deletion may aggravate fatty liver formation through upregulation of SREBP1c.

We also analyzed hepatic expression of a series of pro-inflammatory cytokines and chemokines (Figure 7B). We found that chemokines (including CCR2 and F4/80) and a number of proinflammatory cytokines (including TNF-α, IFN-γ, IL-1β, IL-6, MCP-1, MIP1α, and MIP1β) were significantly increased by ethanol feeding in wild type mice, confirming that ethanol is able to successfully induced inflammatory response in the liver. We also found that Smad7 deletion led to significant increase of chemokines (including CCR2 and F4/80) and proinflammatory cytokines (including TNF-α, IFN-γ, IL-1β, IL-6, MCP-1, MIP1α, and MIP1β. These data indicate that deletion of Smad7 is associated with an elevation of inflammatory response in the liver, likely contributing to the observed hepatic dysfunction in Smad7-deleted mice in this study. Furthermore, the ethanol-induced expression of F4/80, IFN-γ and IL-6 was further elevated by Smad7 deletion, indicating that changes of these factors may underlie the aggravated liver dysfunction in Smad7-deleted mice upon ethanol administration.

## Discussion

In this study, we established a mouse model with liver-specific deletion of Smad7. We found that functional loss of Smad7 in the liver is associated with hyperactivity of TGF-β signaling, as TGF-β1-induced Smad2/3 phosphorylation and EMT was significantly enhanced by Smad7 deletion (Figure 1 and Figure 4). The Smad7^liver-KO^ mice with high efficiency of Smad7 deletion had spontaneous liver dysfunction, demonstrated as general deterioration of the body condition and increased serum levels of AST and ALT (Figure 2), accompanied by liver degeneration and an increase in hepatocyte apoptosis (Figure 3). Furthermore, hepatic injury and steatosis induced by chronic alcohol exposure were accelerated by Smad7 deletion (Figure 5). These data, therefore, reveal for the first time that loss of endogenous Smad7 in the liver can result in spontaneous liver dysfunction and enhance ethanol-induced liver injury.

Our results are consistent with a few recent studies pinpointing the functional role of Smad7 in liver diseases. Overexpression of Smad7 in mouse liver could attenuate TGF-β signaling and TGF-β-induced EMT, while improve CCl_4_-provoked liver fibrosis [19]. On the other hand, hypomorphic Smad7 deficiency could enhance CCl_4_-induced liver damage and fibrosis [20]. The liver damage imposed by Smad7 deletion as observed in this study and by Hamzavi, *et al* is likely mediated by hyperactivity of TGF-β signaling, as overexpression of TGF-β1 specifically in mouse liver leads to increases in hepatic fibrosis and hepatocyte apoptosis [27]. However, unlike this study, spontaneous liver dysfunction was not observed with hypomorphic Smad7 deficiency [20]. We speculate that the difference is dependent on the magnitude of Smad7 deletion. Deletion of the MH1 domain of Smad7 gene only leads to partial loss of Smad7 function [18]. In our study, we found that spontaneous liver dysfunction only occur in Smad7^liver-KO^ mice with high degree of Smad7 deletion (Figure 2). It is speculated that the function of Smad7 needs to be lost to certain degree to initiate spontaneous liver damage in the mouse.

TGF-β is considered one of the most important growth factors that induce EMT process [22], [28], [29]. Activated Smad proteins upon binding of TGF-β to its receptors act as transcription factors to induce expression of EMT-inducing transcription factors within the Snail, ZEB and bHLH families [29]. It has been reported that EMT plays a critical role in the repair of liver tissues after damage and the pathogenesis of liver fibrosis [30]. In this study, we found that loss of Smad7 is associated with enhancement of TGF-β-induced EMT in hepatocytes, consistent with the idea that Smad7 deletion is associated with hyperactivity of TGF-β signaling. Our results are also consistent with the report demonstrating that partial loss of Smad7 function can increase EMT in the liver and accelerates CCl_4_-induced liver fibrosis [20]. It is also in agreement with the finding that overexpression of Smad7 in the liver can inhibit EMT and attenuate TGF-β-mediated fibrogenesis [19]. However, it is noteworthy that whether hepatocytes can directly convert into mesenchymal cells is still controversial. Recently, it was reported that neither hepatocytes nor cholangiocytes could undergo EMT and contribute to liver fibrosis *in vivo* [31], [32].

It is well known that liver is the major organ for detoxification of many chemicals including alcohol. It was recently found that there exist a functional interplay between alcohol-induced liver damage and TGF-β signaling [24]. TGF-β is induced in the mouse liver upon chronic alcohol administration. Interestingly, TGF-β is able to downregulate a key alcohol metabolizing enzyme ADH1 and it is proposed that TGF-β imposes its pro-steatotic action by decreasing the expression of ADH1 in the liver [24]. In our study, we also found that loss of Smad7 function is accompanied by enhancement of alcoholic hepatic steatosis (Figure 5), further indicating the importance of TGF-β signaling in the development of alcohol-induced liver damage. Furthermore, we found that Smad7 deletion is associated with significant reduction of alcohol-induced ADH1 expression in the liver (Figure 6), underscoring the importance of ADH1 in mediating liver injury imposed by hyperactivity of TGF-β signaling.

In addition to the regulation of ADH1 by Smad7 in the liver, we also found that ethanol treatment and Smad7 deletion had evident effects on the expression of a series of genes involved in lipogenesis and inflammatory response (Figure 7). On the one hand, ethanol administration was able to significantly elevate the expression of SREBP1c and its target genes involved in fatty acid synthesis (Figure 7A). This finding is consistent with the notion that SREBP1c is a key regulator of fatty acid synthesis and implicated in the development of fatty liver [25], [26]. Interestingly, the expression of SREBP1c and its target genes involved in fatty acid synthesis was also elevated by Smad7 deletion, indicating that Smad7 deletion may aggravate fatty liver development through upregulation of SREBP1c. On the other hand, we observed that the expression of a series of inflammation-related genes was altered by ethanol treatment and Smad7 deletion (Figure 7B). Chemokines (including CCR2 and F4/80) and a number of proinflammatory cytokines (including TNF-α, IFN-γ, IL-1β, IL-6, MCP-1, MIP1α, and MIP1β) were all elevated by either ethanol treatment or Smad7 deletion. Furthermore, ethanol treatment and Smad7 deletion had a synergistic effect to induce expression of F4/80, IFN-γ and IL-6, indicating that these factors may underlie the aggravated liver dysfunction in Smad7-deleted mice upon ethanol administration. Combining these results, we propose that the alteration of ethanol metabolism, lipogenesis and inflammatory response caused by Smad7 deletion may act together to contribute to severe alcoholic liver injury and steatosis in Smad7-deleted mice. In this regard, our model of liver-specific deletion of Smad7 can serve as a useful tool to comprehend the biological function of endogenous Smad7 in the liver as well as in liver diseases.

## Materials and Methods

### Generation of liver-specific Smad7 deletion mice and genotyping

All animal procedures and protocols were approved by the Institutional Animal Care and Use Committee of the Institute for Nutritional Sciences, Chinese Academy of Sciences with approval number 2010-AN-8. Smad7^loxP/loxP^ mice were developed as previously described [17]. Liver specific deletion of Smad7 mice (Smad7^liver-KO^) were generated by crossing Smad7^loxP/loxP^ mice with Alb-Cre mice that contain a *Cre* recombinase driven by albumin promoter (the Jackson Laboratory, Bar Harbor, ME, USA). Tail biopsies of the mice were analyzed by genomic PCR. The presence of Smad7-loxP allele was detected by primer A (5′-TGTCCCGCTTGTCTTGTTCTTTGAG-3′) and primer G (5′-CAGAGCAGCCGATTGTCTGTTGTGC-3′), resulting in a ∼500-bp PCR product. The wild-type allele was detected by primers A and B (5′-TGCTGACTCTCGTTTCCTGTCTTCG-3′), giving rise to a 154-bp product. The genotyping of Alb-Cre transgenic mouse was performed following the protocol provided by the Jackson Laboratory.

### RNA isolation, RT-PCR and real-time quantitative PCR

Total RNA from mouse tissues was isolated using TRIzol reagent (Invitrogen, Carlsbad, CA, USA). The RNA was treated with RNase-free DNase I and reverse-transcribed with oligo(dT) primer using the SuperScript First-Strand Synthesis System for RT-PCR (Invitrogen). Oligonucleotide primers used for RT-PCR to detect Smad7 mRNA were: 5′-AAGTGTTCAGGTGGCCGGATCTCAG-3′ and 5′-ACAGCATCTGGACAGCCTGCAGTTG-3′ for exon 1-3 of Smad7, 5′-CAACTGCAGGCTGTCCAGATGCTGTAC-3′ and 5′-GTAAACCCACACGCCATCCACTTCC-3′ for exon 3–4 of Smad7. Quantitative real-time PCR was done with the SYBR Green PCR system (Applied Biosystems, Foster City, CA, USA), using actin as an internal control for normalization. Primers used for each gene are listed as follows: 5′-CTCCTCCTTTCTCGTCATCC-3′ and 5′- CACACACACAACCCAACAAA-3′ for the mRNA region corresponding to exon 4 of Smad7, 5′-GGCCGCCTTGACACCAT-3′ and 5′-GCACTCCTACGACGACGCTTA-3′ for ADH-1, 5′-GATCATTGCTCCTCCTGAGC-3′ and 5′-ACTCCTGCTTGCTGATCCAC-3′ for β-actin. Other primers used in this study are listed in Table S1.

### Mouse model of chronic-binge ethanol consumption

The chronic alcohol-fed mouse model was established as previously described with minor modification [21], [33]. In brief, 10 to 12 weeks old male mice were fed with a nutritionally adequate liquid diet containing 5% ethanol or a control diet for up to 6 weeks (Dyets, Inc., Bethlehem, PA, USA). Both diets were dispensed in glass liquid-diet feeding tubes. Ethanol was introduced gradually by increasing the content by 1% (vol/vol) every day until the mice were consuming diet containing 5% (vol/vol) ethanol for up to 6 weeks. After that, the mice of ethanol-treated group were gavaged with a single doses of ethanol (5 g/kg body weight, 10% ethanol), whereas mice in control groups were gavaged with isocaloric dextrin maltose. After gavage, mice were kept on control or ethanol diet and euthanized 6 hours later.

### Isolation of primary mouse hepatocytes

Mouse hepatocytes were isolated from livers of 8-week-old mice by a modified two-step collagenase perfusion protocol [34]. In brief, the hepatocytes were plated on collagen I coated 6-well plates (3×10^5^ cells/well) in Dulbecco modified Eagle medium-F-12 (DMEM, from GIBCO-BRL, Gaithersburg, MD, USA) with supplements as described previously [35]. The medium was changed after 4 h with DMEM supplemented with 1% penicillin/streptomycin. For TGF-β1 treatment, the cell culture medium was changed to serum-free DMEM with 1% penicillin/streptomycin and 5 ng/ml TGF-β1 (Sigma–Aldrich, St. Louis, MO, USA) was added as indicated. For ethanol treatment, 100 mmol/L ethanol was added in fresh medium for 24 hours. The plates were sealed with parafilm to prevent evaporation after the addition of ethanol.

### Cell motility assay

Primary hepatocytes were plated on collagen I-coated 6-well plates (3×10^5^ cells/well) and then “wounded” by scratching the cells with a 200 µl pipette tip in the presence or absence of TGF-β1 after attachment and then monitored in 24 and 48 h by phase-contrast microscope photography as described previously [23].

### Immunoblotting analysis

For Western blotting analysis, the cells and tissues were lysed in a radioimmunoprecipitation assay (RIPA) buffer (150 mmol/L NaCl, 1% Triton X-100, 0.5% sodium deoxycholate, 0.1% SDS, 50 mmol/L Tris-HCl at pH 7.4) containing phosphatase inhibitors and a protease inhibitor cocktail (Sigma-Aldrich). The lysate was subjected to SDS-PAGE, transferred to polyvinylidene fluoride (PVDF) membranes, and incubated with the primary antibodies, followed by horseradish peroxidase-conjugated secondary antibody (Amersham, Little Chalfont, Bucks, UK). The bound antibody was visualised using enhanced chemiluminescence reagents (Pierce, Rockford, USA). The antibodies used were as follows: rabbit anti-actin antibody (Santa Cruz Biotechnology, Santa Cruz, CA, USA), goat anti-Smad7 antibody (Santa Cruz Biotechnology), rabbit anti-phosphorylated-Smad2 antibody (Cell Signaling Technology, Beverly, MA, USA), rabbit anti-phosphorylated-Smad3 antibody (Cell Signaling Technology), goat anti-Smad2/3 antibody (Santa Cruz Biotechnology), rabbit anti-cleaved-caspase-3 antibody (Cell Signaling Technology), mouse anti-E-cadherin antibody (BD Transduction Laboratories, New Jersey, USA), and mouse anti-vimentin antibody (Santa Cruz Biotechnology).

### Analysis of blood and tissue samples

The serum levels of alanine transaminase (ALT), aspartate transaminase (AST) and total cholesterol (TC) were determined by a kit from ShenSuoYouFu (Shanghai, China). Triglycerides were determined by the Serum Triglyceride Determination Kit (Sigma-Aldrich). The measurement of hepatic triglyceride was following a protocol as previously reported [36]. About 40–60 mg of liver tissue was homogenized in a total of 4 ml of a mixture of CHCl_3_-CH_3_OH (2∶1, v/v). After addition of 1 ml 0.88% NaCl, the homogenate was centrifuged at 3700 rpm for 10 min at room temperature. The portion at the lower organic phase was transferred to a new tube and dried under nitrogen. The dried residue was resuspended in 1 ml of 1% Triton X-100 in absolute ethanol.

### Histology and immunohistochemistry

Following fixation of the livers with 10% formalin/phosphate-buffered saline, paraffin-embedded sections were subjected to standard Hematoxylin & Eosin (H&E) staining. Hepatic lipid content was determined by 10 µm thick frozen sections stained with Oil Red O (Sigma–Aldrich). The immunohistochemistry was performed with 5 µm sections using SABC (mouse/rabbit IgG) kit according to the manufacturer's instruction (Boster, Wuhan, Hubei, China). The primary antibodies used were as follows: phosphorylated Smad2 (1∶200, Cell Signaling Technology) and cleaved caspase-3 (1∶1000, Cell Signaling Technology). TUNEL assay was carried out using ApopTagH Peroxidase In Situ Apoptosis Detection Kit (from Chemicon, Temecula, CA, USA) following the manufacturer's instructions.

### Annexin V staining

Hepatocytes were plated on coverslips (∼80,000 cells per well in 6 well plates). After overnight serum starvation, cells were treated with 5 ng/mL TGF-β1 as indicated. Residual culture medium was washed off the cells with phosphate-buffered saline. Cells were stained for 5 minutes with Annexin V-PE Apoptosis Detection Kit (BioVision Inc., Mountain View, CA, USA) and 5 µg/mL Hoechst 33342 (Molecular Probes, Eugene, OR, USA) in phosphate-buffered saline. Unbound stain was washed off the cells with phosphate-buffered saline, and fluorescent signal was detected immediately.

### Immunofluorescence

Immunofluorescence staining was performed as described previously [37]. Fluorescein isothiocyanate-phalloidin (Sigma-Aldrich) was used to detect F-actin. Cell nuclei were counterstained with Hoechst 33342.

### Statistical analysis

Statistically significance was assessed by one-way ANOVA or Student's t test.

## Supporting Information

Figure S1 **Expression of Cre recombinase in the wild type and Smad7liver-KO mice.** The liver samples as for Figure 2B were used to determine the mRNA level of Cre recombinase by real-time PCR. The relative mRNA levels of Smad7 (exon4, also shown in Figure 2B) and Cre are shown. Please note that in general high expression of Cre is associated with low expression of Smad7.
(DOC)

Table S1 **Primer sequences for mouse genes used in real-time PCR.**
(DOC)

## References

[1] Murray KF, Hadzic N, Wirth S, Bassett M, Kelly D (2008). Drug-related hepatotoxicity and acute liver failure.. J Pediatr Gastroenterol Nutr.

[2] Castilla A, Prieto J, Fausto N (1991). Transforming growth factors beta 1 and alpha in chronic liver disease. Effects of interferon alfa therapy.. N Engl J Med.

[3] Massague J (1998). TGF-beta signal transduction.. Annu Rev Biochem.

[4] Heldin CH, Miyazono K, ten Dijke P (1997). TGF-beta signalling from cell membrane to nucleus through SMAD proteins.. Nature.

[5] Miyazono K, Kusanagi K, Inoue H (2001). Divergence and convergence of TGF-beta/BMP signaling.. J Cell Physiol.

[6] Wrana JL, Attisano L, Carcamo J, Zentella A, Doody J (1992). TGF beta signals through a heteromeric protein kinase receptor complex.. Cell.

[7] Hayashi H, Abdollah S, Qiu Y, Cai J, Xu YY (1997). The MAD-related protein Smad7 associates with the TGFbeta receptor and functions as an antagonist of TGFbeta signaling.. Cell.

[8] Weinstein M, Yang X, Deng C (2000). Functions of mammalian Smad genes as revealed by targeted gene disruption in mice.. Cytokine Growth Factor Rev.

[9] Tremblay KD, Dunn NR, Robertson EJ (2001). Mouse embryos lacking Smad1 signals display defects in extra-embryonic tissues and germ cell formation.. Development.

[10] Waldrip WR, Bikoff EK, Hoodless PA, Wrana JL, Robertson EJ (1998). Smad2 signaling in extraembryonic tissues determines anterior-posterior polarity of the early mouse embryo.. Cell.

[11] Takaku K, Oshima M, Miyoshi H, Matsui M, Seldin MF (1998). Intestinal tumorigenesis in compound mutant mice of both Dpc4 (Smad4) and Apc genes.. Cell.

[12] Zhu Y, Richardson JA, Parada LF, Graff JM (1998). Smad3 mutant mice develop metastatic colorectal cancer.. Cell.

[13] Yang X, Letterio JJ, Lechleider RJ, Chen L, Hayman R (1999). Targeted disruption of SMAD3 results in impaired mucosal immunity and diminished T cell responsiveness to TGF-beta.. EMBO J.

[14] Chang H, Huylebroeck D, Verschueren K, Guo Q, Matzuk MM (1999). Smad5 knockout mice die at mid-gestation due to multiple embryonic and extraembryonic defects.. Development.

[15] Huang Z, Wang D, Ihida-Stansbury K, Jones PL, Martin JF (2009). Defective pulmonary vascular remodeling in Smad8 mutant mice.. Hum Mol Genet.

[16] Galvin KM, Donovan MJ, Lynch CA, Meyer RI, Paul RJ (2000). A role for smad6 in development and homeostasis of the cardiovascular system.. Nat Genet.

[17] Chen Q, Chen H, Zheng D, Kuang C, Fang H (2009). Smad7 is required for the development and function of the heart.. J Biol Chem.

[18] Li R, Rosendahl A, Brodin G, Cheng AM, Ahgren A (2006). Deletion of exon I of SMAD7 in mice results in altered B cell responses.. J Immunol.

[19] Dooley S, Hamzavi J, Ciuclan L, Godoy P, Ilkavets I (2008). Hepatocyte-specific Smad7 expression attenuates TGF-beta-mediated fibrogenesis and protects against liver damage.. Gastroenterology.

[20] Hamzavi J, Ehnert S, Godoy P, Ciuclan L, Weng H (2008). Disruption of the Smad7 gene enhances CCI4-dependent liver damage and fibrogenesis in mice.. J Cell Mol Med.

[21] Horiguchi N, Wang L, Mukhopadhyay P, Park O, Jeong WI (2008). Cell type-dependent pro- and anti-inflammatory role of signal transducer and activator of transcription 3 in alcoholic liver injury.. Gastroenterology.

[22] Kalluri R, Weinberg RA (2009). The basics of epithelial-mesenchymal transition.. J Clin Invest.

[23] Cano A, Perez-Moreno MA, Rodrigo I, Locascio A, Blanco MJ (2000). The transcription factor snail controls epithelial-mesenchymal transitions by repressing E-cadherin expression.. Nat Cell Biol.

[24] Ciuclan L, Ehnert S, Ilkavets I, Weng HL, Gaitantzi H (2010). TGF-beta enhances alcohol dependent hepatocyte damage via down-regulation of alcohol dehydrogenase I. J Hepatol.

[25] Horton JD, Goldstein JL, Brown MS (2002). SREBPs: activators of the complete program of cholesterol and fatty acid synthesis in the liver.. J Clin Invest.

[26] Ji C, Chan C, Kaplowitz N (2006). Predominant role of sterol response element binding proteins (SREBP) lipogenic pathways in hepatic steatosis in the murine intragastric ethanol feeding model.. J Hepatol.

[27] Sanderson N, Factor V, Nagy P, Kopp J, Kondaiah P (1995). Hepatic expression of mature transforming growth factor beta 1 in transgenic mice results in multiple tissue lesions.. Proc Natl Acad Sci U S A.

[28] Miettinen PJ, Ebner R, Lopez AR, Derynck R (1994). TGF-beta induced transdifferentiation of mammary epithelial cells to mesenchymal cells: involvement of type I receptors.. J Cell Biol.

[29] Xu J, Lamouille S, Derynck R (2009). TGF-beta-induced epithelial to mesenchymal transition.. Cell Res.

[30] Choi SS, Diehl AM (2009). Epithelial-to-mesenchymal transitions in the liver.. Hepatology.

[31] Taura K, Miura K, Iwaisako K, Osterreicher CH, Kodama Y (2010). Hepatocytes do not undergo epithelial-mesenchymal transition in liver fibrosis in mice.. Hepatology.

[32] Scholten D, Osterreicher CH, Scholten A, Iwaisako K, Gu G (2010). Genetic labeling does not detect epithelial-to-mesenchymal transition of cholangiocytes in liver fibrosis in mice.. Gastroenterology.

[33] Ki SH, Park O, Zheng M, Morales-Ibanez O, Kolls JK (2010). Interleukin-22 treatment ameliorates alcoholic liver injury in a murine model of chronic-binge ethanol feeding: role of signal transducer and activator of transcription 3.. Hepatology.

[34] Neufeld DS (1997). Isolation of rat liver hepatocytes.. Methods Mol Biol.

[35] Bottinger EP, Factor VM, Tsang ML, Weatherbee JA, Kopp JB (1996). The recombinant proregion of transforming growth factor beta1 (latency-associated peptide) inhibits active transforming growth factor beta1 in transgenic mice.. Proc Natl Acad Sci U S A.

[36] Yang L, Zhang Y, Wang L, Fan F, Zhu L (2010). Amelioration of high fat diet induced liver lipogenesis and hepatic steatosis by interleukin-22.. J Hepatol.

[37] Zavadil J, Cermak L, Soto-Nieves N, Bottinger EP (2004). Integration of TGF-beta/Smad and Jagged1/Notch signalling in epithelial-to-mesenchymal transition.. EMBO J.

