# Supplementary figures and images for: Hepatic Deletion of Smad7 in Mouse Leads to Spontaneous Liver Dysfunction and Aggravates Alcoholic Liver Injury
Source: PLoS One. 2011 Feb 28;6(2):e17415. doi: 10.1371/journal.pone.0017415 (PMC3046253; doi:10.1371/journal.pone.0017415)

**Figure S1. Expression of Cre recombinase in the wild type and Smad7liver-KO mice**


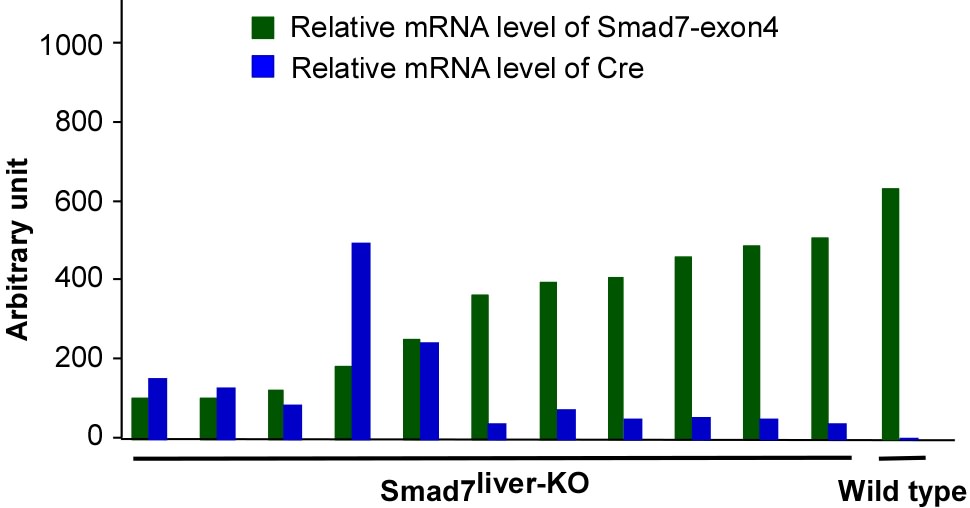

Supplement: Figure S1 — Expression of Cre recombinase in the wild type and Smad7liver-KO mice. The liver samples as for Figure 2B were used to determine the mRNA level of Cre recombinase by real-time PCR. The relative mRNA levels of Smad7 (exon4, also shown in Figure 2B) and Cre are shown. Please note that in general high expression of Cre is associated with low expression of Smad7. (DOC) [file pone.0017415.s001.doc]
