# Supplementary material for: Hepatic Deletion of Smad7 in Mouse Leads to Spontaneous Liver Dysfunction and Aggravates Alcoholic Liver Injury
Source: PLoS One. 2011 Feb 28;6(2):e17415. doi: 10.1371/journal.pone.0017415 (PMC3046253; doi:10.1371/journal.pone.0017415)
Supplement: Table S1 — Primer sequences for mouse genes used in real-time PCR. (DOC) [file pone.0017415.s002.doc]

**Table S1. Primer sequences for mouse genes used in real-time PCR**

| **Genes** | **Forward primer (5’ to 3’)** | **Reverse primer (5’ to 3’)** |
| --- | --- | --- |
| **ADH-1** | GATCATTGCTCCTCCTGAGC | ACTCCTGCTTGCTGATCCAC |
| **TNF-α** | AAGCCTGTAGCCCACGTCGTA | AGGTACAACCCATCGGCTGG |
| **F4/80** | GGAAAGCACCATGTTAGCTGC | CCTCTGGCTGCCAAGTTAATG |
| **CCR2** | ATGCAAGTTCAGCTGCCTGC | ATGCCGTGGATGAACTGAGG |
| **IL-1β** | AAAAAAGCCTCGTGCTGTCG | GTCGTTGCTTGGTTCTCCTTG |
| **IL-6** | TCCATCCAGTTGCCTTCTTG | TTCCACGATTTCCCAGAGAAC |
| **IFN-γ** | TAGCCAAGACTGTGATTGCGG | AGACATCTCCTCCCATCAGCAG |
| **MCP-1** | TCAGCCAGATGCAGTTAACGC | TCTGGACCCATTCCTTCTTGG |
| **MIP-1α** | TGCCCTTGCTGTTCTTCTCTG | CAACGATGAATTGGCGTGG |
| **MIP-1β** | AACACCATGAAGCTCTGCGTG | TGTCTGCCTCTTTTGGTCAGG |
| **SREBP1c** | GAGGCCAAGCTTTGGACCTGG | CCTGCCTTCAGGCTTCTCAGG |
| **FAS** | GGAGGTGGTGATAGCCGGTAT | TGGGTAATCCATAGAGCCCAG |
| **ACC1** | TGAAGGGCTACCTCTAATG | TCACAACCCAAGAACCAC |
| **SCD1** | CTGCCTCTTCGGGATTTTCTACT | GCCCATTCGTACACGTGATTC |
